# Anticancer activity and molecular docking of active ingredients from ethanolic extract of grapefruit peels against HepG2 cell

**DOI:** 10.1038/s41598-026-56458-w

**Published:** 2026-06-10

**Authors:** Emad A. Shalaby, Ahmed M. Aboul-Enein, Mariam M. Soliman, Soha S. Mostafa, Nourhan A. Aboelnaga, Clara R. Azzam

**Affiliations:** 1 Department of Biochemistry, Faculty of Agriculture, Cairo University, Giza, 12613 Egypt; 2 Faculty of Agriculture, Biotechnology Program, Cairo University, Giza, 12613 Egypt; 3 Department of Microbiology, Soils, Water and Environment Research Institute, ARC, Giza, 12619 Egypt; 4 Genetics Department, Faculty of Agaric, Ain Shams University, P.O. Box 68, Hadayek Shoubra, 11241 Cairo, Egypt; 5 Department of Cell Research, Field Crops Research Institute, ARC, Giza, 12619 Egypt

**Keywords:** Natural products, Grapefruit peel, Viability, Naringenin, HepG2 cell line, Biochemistry, Biotechnology, Cancer, Computational biology and bioinformatics, Drug discovery, Plant sciences

## Abstract

Natural products are vital sources for drug discovery, offering greater structural diversity than synthetic chemicals. They have historically contributed to the identification of bioactive molecules and continue to play a crucial role in the discovery of new therapeutics. *Citrus* species, members of the *Rutaceae* family, represent one of the most economically important fruit tree crops worldwide, with a global yield of approximately 123 million tons in 2010. grapefruit peels were collected, dried and their active ingredients were extracted with 70% ethanol. The resulting extracts underwent phytochemical screening. The anticancer activity of the extracts was evaluated using the MTT assay, with cell viability assessed and IC₅₀ values determined. Promising fractions were further analyzed using high-performance liquid chromatography (HPLC). **Results** Phytochemical screening revealed high phenolic and flavonoid content, with naringenin being the most abundant compound, followed by chlorogenic acid and ferulic acid. The extract exhibited significant anticancer activity against HepG2 cells, with an IC_50_ of 230.63 *±* 1.61 µg/ml. Molecular docking analysis of sixteen phytochemicals against the AFP protein (PDB ID: 7YIM) identified several compounds with strong binding affinities toward the predicted active site. Ellagic acid showed the highest binding affinity (-8.9 kcal/mol), followed by rutin (-8.8 kcal/mol), quercetin (-8.5 kcal/mol), and naringenin (-8.4 kcal/mol). Interaction analysis revealed multiple hydrogen bonding and π-interactions with key AFP residues, including LYS228, CYS224, PHE172, GLU489, and MET490. These findings highlight polyphenolic compounds, particularly ellagic acid and rutin, as promising lead molecules for AFP-targeted anti-hepatocellular carcinoma therapy. Grapefruit peel extract exhibits promising antioxidant and anticancer activities, demonstrating concentration-dependent cytotoxicity against HepG2 cells. Despite its lower potency compared to doxorubicin, it represents a valuable natural source of bioactive compounds. Further studies are required to elucidate its mechanisms, safety, and potential application in hepatocellular carcinoma therapy.

## Introduction

Hepatocellular carcinoma (HCC) accounts for over 90% of all primary liver cancers and is the sixth most common cancer worldwide, with significant mortality, particularly in regions with high hepatitis prevalence, such as Egypt. After lung cancer, it is the second leading cause of male cancer death. Significant risk factors for hepatocellular cancer include viral hepatitis (hepatitis B and C), alcoholic liver disease, and non-alcoholic liver steatohepatitis/non-alcoholic fatty liver disease^1^.

Egypt has a high HCV transmission rate, with around 416,000 new cases each year^2^. There is evidence of a link between HCV and improved HCC outcomes. The number of confirmed cases of both diseases rose as a result of the screening and follow-up program initiated by the government. According to the study by Ziada et al.^3^, ultrasound-detected localized lesions were found in 108 (21%) of 514 HCV-infected patients. HCC occurred more frequently in HCV-infected individuals than in HBV-infected ones^4^. These discoveries could highlight the essential gambling factor for advancing HCC in Egypt. The Global Cancer Observatory found that carcinoma of the liver was the cause of 19% of all newly diagnosed cases in 2018 among people of all ages and genders, with a 32% incidence rate and a 31% mortality rate^5^. Nowadays, natural products are a significant source of drug improvement and have a more diverse variety in structure on a greater scale than blended synthetic compounds. Natural products have been used for the treatment of several diseases as an alternative medicine and will continue to be important in the identification of new medications^6^.

*Citrus* belongs to the *Rutaceae* family and is the world’s most critical natural product tree crop, with a yearly yield of around 123 million tons in 2010. Bioactive chemicals are abundant in *Citrus* fruits like grapefruit. *Citrus* fruits contain a wide range of bioactive compounds, including flavonoids, which are mainly concentrated in the peel, albedo (white inner layer), and, to a lesser extent, the pulp. They are also rich in nutrients such as folate, vitamin C, and dietary fiber. Flavonoids are widely distributed in higher plants, but citrus species are particularly rich in glycosylated flavanones. In citrus, the predominant flavonoids occur as glycosides, mainly hesperidin and naringin. All grapefruit varieties belong to the species *Citrus × paradisi*. As reported by Peterson et al.^7^, variation in pulp color among cultivars is primarily attributed to the presence of lycopene, which imparts a red pigmentation, whereas its absence results in white-fleshed varieties. Grapefruit is widely consumed globally and is commonly included in dietary practices across different populations^8^. Recent evidence reported by Li et al.^9^ highlighted that naringenin, a major flavonoid constituent of grapefruit, exhibits significant anti-angiogenic and chemopreventive properties. Furthermore, naringenin has been shown to exert protective effects against hepatocellular carcinoma by inducing apoptosis, downregulating key growth factors such as TGF-β and VEGF, and modulating MAPK signaling pathways. Overall, naringenin is considered a bioactive and relatively safe compound with multi-targeted molecular actions^10^.

Several natural compounds have been reported to exert hepatoprotective effects, among which naringenin is one of the most important flavonoids widely distributed in plants, particularly in grapefruits. Naringenin exhibits strong antioxidant, anti-inflammatory, and anti-angiogenic activities, and has been shown to induce apoptosis in various cancer cell lines, including HepG2 hepatocellular carcinoma cells. Its hepatoprotective effects are mainly associated with the modulation of oxidative stress, regulation of the TGF-β signaling pathway, and inhibition of hepatic stellate cell activation and trans-differentiation, thereby reducing collagen deposition and liver fibrosis. In addition, naringenin demonstrates notable anticancer potential through its ability to induce apoptosis, suppress angiogenesis, and arrest the cell cycle in different cancer models, including human hepatocellular carcinoma cells^9^.

The present study aimed to evaluate the anticancer activity of ethanolic grapefruit peel extract against HepG2 cells, identify its major bioactive constituents using HPLC analysis, and investigate their potential molecular interactions through in silico docking studies.

## Materials and methods

### Chemicals

The Sigma-Aldrich company supplied the high-purity chemicals and reagents used in this study (DPPH, Doxorubicin, ABTS, Ascorbic acid, Folin reagent, TLC silica gel F_254_).

### Extraction of active ingredients

During the spring of 2023, the Grapefruit peel plant was collected and purchased from a local market in Cairo, Egypt. After being collected, the biomass was dried in the air and ground into a fine powder. According to Rosenthaler^11^, the dried powder (10 g) underwent individual extraction using 70% ethanol as the solvent. The organic solvent extract was concentrated in a water bath at temperatures ranging from 40 to 50 °C. Qualitative phytochemical screening was performed for phenolics, flavonoids, alkaloids, glycosides, anthocyanins, coumarins, reduced sugars, starch, proteins and fatty acids using standard methods^12–16^.

### Phytochemical screening

The ethanolic grapefruit peel extract was subjected to qualitative phytochemical screening to identify its major bioactive constituents using standard analytical procedures. Carbohydrates and reducing sugars were detected using Molisch’s test and Fehling’s reagent, respectively, as described in classical phytochemical protocols. The presence of amino acids and proteins was determined according to the method of Shaikh and Patil^12^, while alkaloids were identified using Wagner’s test^12^.

Phenolic compounds were detected using ferric chloride reagent following the method described by Shaikh and Patil^12^. Flavonoids were screened using the procedure of Wall et al.^13^, whereas anthocyanins and coumarins were identified according to Sawant and Godghate^14^. Fatty acids were analyzed using the method described by Abdulkadir and Tsuchiya^16^.

In addition, antioxidant activity was evaluated using a qualitative potassium permanganate (KMnO₄) decolorization assay, as reported by Gaber et al.^15^, to assess free radical scavenging potential.

### Determination of total phenolic compounds

Singleton and Rossi’s^17^ method was used to identify phenolic compounds. 100 mL of the extract and 750 mL of the Folin-Ciocalteu reagent (10%) were combined. A saturated sodium carbonate solution (6%) of 750 µl was added to the mixture after three minutes. The response combination was incubated in the dark (inside a dark cabinet) for 1.5 h.The absorbance was estimated at 725 nm utilizing a spectrophotometer. Phenolic contents were determined in light of the standard curve of Gallic corrosive utilized as a standard.

### Determination of total flavonoids

Flavonoid compounds were resolved utilizing the technique depicted by Zhishen et al.^18^. 125 µl of the concentrate was blended with 150 µl AlCl_3_ (10%, w/v). The reaction was incubated for 15 min in the dark with 750 µl of 1 M NaOH added after 5 min. The absorbance was measured using spectrophotometry against a blank at 510 nm after the mixture was thoroughly mixed. When preparing the calibration curve, quercetin was used as the standard compound.

### Determination of total catechins

The vanillin assay, as described by Khlifi et al.^19^, was used to measure the concentrated tannins in the plant extracts, as well as Belyagoubi-benhammou et al.^20^, with a few alterations. 1500 mL of a vanillin/methanol (4%) solution was mixed with 50 mL of that extract. The absorbance at 550 nm was measured against a blank after 750 mL of concentrated HCl was added and allowed to react at room temperature for one hour. The complete centralization of dense tannins was communicated in micrograms of catechin counterparts per milligram dry matter regarding the catechin adjustment curve.

### Thin-layer chromatography (TLC)

The separation of active compounds from Grapefruit peel extracts was performed using Precoated silica gel plates (TLC F_254_) with different solvent systems as mobile phases, as follows: Hexane: acetone and Ethyl acetate: ethanol at a ratio of 9:1 (v/v).

A rapid TLC screening method for antioxidant activity was performed using the 2, 2-diphenyl-1-picryl-hydrazyl radical (DPPH) as a spray reagent. TLC was performed for Grapefruit peel extracts. The plates were dried, and antioxidant activity was detected by spraying 0.2% 2, 2-diphenyl-2-picrylhydrazyl (DPPH) onto TLC plates. TLC was performed for hexane, ethyl acetate, and ethanol 70% extracts as described earlier by Nair et al.^21^. The development of yellow or white spots against a purple background indicates the presence of an antioxidant compound.

### Determination of ascorbic acid in water extract

One mL of the plant extract solution was transferred into a test tube and then titrated with 0.1% Dichlorophenol Indophenol (DCPIP) solution dropwise until the initial appearance of blue color. DCPIP was added until the blue color didn’t disappear, and the volume of DCPIP used was calculated. This volume is equivalent to the amount of ascorbic acid present in the extract, so the concentration of ascorbic acid in the original plant material can be calculated and expressed as units per gram of fresh mass from a previous titration of the dye with standard vitamin C^22^. Hence, each mL of 0.1% DCPIP (Mw 290.08) solution is equivalent to 6.071 × 10^− 4^g ascorbic acid (Mw 176.12). Therefore, 1 cm³ of extract must contain 9.107 × 10^− 4^g of ascorbic acid.

### High-pressure liquid chromatography (HPLC)

HPLC separation was performed using an Agilent 1260 system with a C18 column (4.6 mm x 250 mm I.D., 5 μm). The mobile phase consisted of water (A) and acetonitrile (B) with 0.05% trifluoroacetic acid (TFA), with a linear gradient over minutes at 0.9 mL/min. The mobile phase was programmed consecutively in a linear gradient as follows: 0 min (82% A); 0–5 min (80% A); 5–8 min (60% A); 8–12 min (60% A); 12–15 min (82% A); 15–16 min (82% A) and 16–20 (82%A). The multi-wavelength detector was monitored at 280 nm. The injection volume was 5 µl for each of the sample solutions, and the column temperature was maintained at 40 °C.

### Pigment analysis: extraction and determination of chlorophylls and carotenoids

The extraction and quantification of chlorophylls and carotenoids were performed according to the method described by Holden^23,24^. After pigment extraction, the apparent color was measured at 663, 645 and 452 nm, respectively, in a 1 cm quartz cell, against a blank. The following equation was used for calculating the concentration of each chlorophyll and carotenoid (as mg/g):

Chlorophyll a.

Chlorophyll a (mg/g) = ((12.3*A663–0.8*A645) * V)/(A * 100 * W).

Chlorophyll b.

Chlorophyll b (mg/g) = ((19.3*A645–3.6*A663) * V)/(A * 100 * W).

Total carotenoids.

Total carotenoids (mg/g) = 4.57*A452–0.22*(Total chlorophyll).

Where: A_645_ and A_663_ are the absorbances at A_645_ and A_663_ nm; V is the volume in mL; A is the length of the light path in the cell; and W is the fresh weight in grams.

### Viability assay

#### Determination of grapefruit peel extracts cytotoxicity on HepG2 cells (MTT protocol)

The cytotoxicity of Grapefruit peel extracts was evaluated against HepG2 cells using the MTT protocol according to Hansen et al.^25^, as follows: The 96-well tissue culture plate was inoculated with 1 × 10^˄^5 cells/mL (100 µl/well) and incubated at 37 °C for 24 h to develop a complete monolayer sheet. The growth medium was decanted from 96-well microtiter plates after the confluent sheet of HepG2 cells was formed. The cell monolayer was washed twice with wash media, and two-fold dilutions of the tested sample were made in RPMI medium with 2% serum (maintenance medium). 0.1 mL of each dilution was tested in different wells, leaving 3 wells as controls, receiving only maintenance medium. The plate was incubated at 37 °C and then examined. HepG2 Cells were checked for any physical signs of toxicity, e.g., partial or complete loss of the monolayer, rounding, shrinkage, or cell granulation. MTT solution was prepared (5 mg/mL in PBS) (BIO BASIC CANADA INC), and 20 µl MTT solution was added to each well. Placed on a shaking table, 150 rpm for 5 min, to thoroughly mix the MTT into the media. The plate was incubated (37 °C, 5% CO_2_) for 4 h to allow the MTT to be metabolized and removed from the media. The formazan produced from the MTT (MTT metabolic product) was resuspended in 200 µl DMSO, and placed on a shaking table, 150 rpm for 5 min, to thoroughly mix the formazan into the solvent. Optical density (OD) was measured at 560 nm, with background correction performed by subtracting the absorbance at 620 nm. The resulting OD values were used as an indicator of cell viability, assuming a direct correlation between optical density and the number of viable cells.

### Molecular docking

Docking was performed using AutoDock 4.2^26^ The three-dimensional structure of Alpha-fetoprotein (AFP; PDB ID: 7YIM) was retrieved from the RCSB Protein Data Bank, prepared using Discovery Studio Visualizer, and its active binding site was predicted using the PrankWeb server. Sixteen phytochemical ligands obtained from PubChem were prepared and docked against AFP using PyRx/AutoDock Vina, while protein–ligand interactions and binding modes were subsequently analyzed and visualized in Discovery Studio Visualizer.

### Statistical analysis

All the data are expressed as mean ± standard deviation. Statistical comparison was performed via a one-way analysis of variance followed by Duncan’s multiple range test^27^. P-values of less than 0.05 (P˂0.05) were considered significant.

## Results and discussion

### Phytochemical screening of ethanolic extracts from grapefruit peels

The preliminary qualitative screening for phytochemicals of ethanolic extracts from *Grapefruit peels* revealed that the Phenolic, Flavonoids and Fatty acids were detected as shown in Table 1.

**Table 1:** Phytochemical screening of ethanolic extracts from grapefruit peels.

Phytochemical compound	Result	Phytochemical compound	Result
Phenolic	+	Anthocyanins	-
Reducing sugars	-	Coumarins	-
Proteins	-	Glycosides	-
Fatty acids	+	Alkaloids	-
Flavonoids	+	Antioxidant activity	-

+: present; -: absent.

These results were in agreement with Oikeh et al.^28^ and El-Beltagi et al.^29^, who showed that phenolic and flavonoid compounds were detected in their Grapefruit peel samples. Flavonoids have a dual activity in terms of ROS homeostasis; under normal conditions, they operate as antioxidants. Also, in cancer cells, they are powerful pro-oxidants, activating apoptotic pathways and downregulating pro-inflammatory signalling pathways^30^. Not only flavonoids have anti-cancer activity, but also many phenolic compounds such as catechins, gallic acid, quercetin, naringenin and rutin acid have therapeutic antioxidant, anti-inflammatory and anti-cancer activity. The undetected compounds may be due to the sample being diluted.

### Phenolic, flavonoid, catechin and ascorbic acid contents

Total phenolic, flavonoid, catechin and ascorbic acid contents (as µg/g) in ethanolic extracts of Grapefruit peels were measured (Table 2). The phenolic compounds were the highest ingredient at 867 ppm, flavonoids at 340 ppm and catechins at 81.8 ppm.

**Table 2:** Phenolic, flavonoid, catechin and ascorbic acid contents (as µg/g, ppm) of ethanolic extract from Grapefruit peels.

Phenolic compounds	Flavonoids	Catechins	Ascorbic acid
**867 ± 16.67**	**340 ± 9.61**	**81.8 ± 1.24**	**26.84 ± 2.80**

Phenolic compounds are beneficial to human health due to their potential antioxidants and prevent damage to cells resulting from free radical oxidation reactions. Also, phenolic compounds are useful as signalling molecules, antidiabetic agents, and antimicrobials^31,32^. The compounds can also limit the generation or activity of pro-inflammatory mediators, giving them anti-inflammatory properties^33^. Our results complied with those of Abotaleb et al.^34^, who clarified that phenolics have a promising future as cytotoxic anti-cancer agents that target angiogenesis, growth, differentiation, and metastasis, as well as promote apoptosis and reduce proliferation. The findings agreed with the results of investigations carried out by Rekha et al.^35^ and Liew et al.^36^.

### Chlorophyll and total carotenoids

The contents of chlorophylls and carotenoids were determined in Grapefruit extracts as outlined in Table 3, which showed that chlorophyll b was higher than chlorophyll a, while total carotenoids were higher than total chlorophylls. The results complied with those of Ling et al.^37^, who illustrated that non-irradiated plantlets demonstrated the highest amount of chlorophyll content as compared to plantlets irradiated with gamma rays. In addition, the amount of chlorophyll a was higher than chlorophyll b in both irradiated and non-irradiated plantlets.

**Table 3:** Chlorophylls and total carotenoids (as mg/g) in ethanolic extract from Grapefruit peels.

Chlorophyll a	Chlorophyll b	Total chlorophylls	Total carotenoids
**0.038**	**0.055**	**0.094**	**2.63**

Plants contain two kinds of chlorophyll: chlorophyll a and b, both possess antioxidant properties and are fat-soluble compounds^38^. The results confirmed that this compound has some positive effects on health. Chlorophyll has been shown in animal studies to lessen the incidence of malignant tumors. It was discovered that chlorophyll may form tight interactions with carcinogenic compounds known as aflatoxins. When they bond, chlorophyll aids in the absorption of aflatoxins (cancer-causing chemicals) in the intestines. The anticancer activity of the tested extracts in the present study may also be related to the occurrence of plant pigments, including chlorophyll and carotenoids. Some studies showed cancer prevention; hence, Chlorophyll helps to prevent DNA damage caused by toxic aflatoxins. Carotenoids have antioxidant qualities and may benefit human health when ingested in sufficient quantities. According to Maoka^39^, chlorophyll and carotenoids are essential for plant function and survival rates.

### Viability of cancer cells

**Fig. 1:**
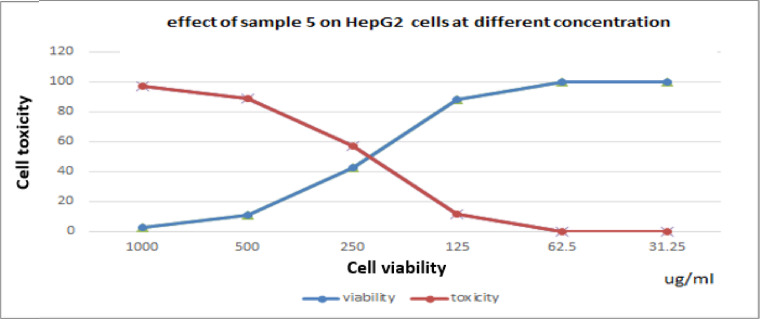
Chart representing the cell viability and cell toxicity of grapefruit peel extract against liver cell lines (HepG2).

**Fig. 2:**
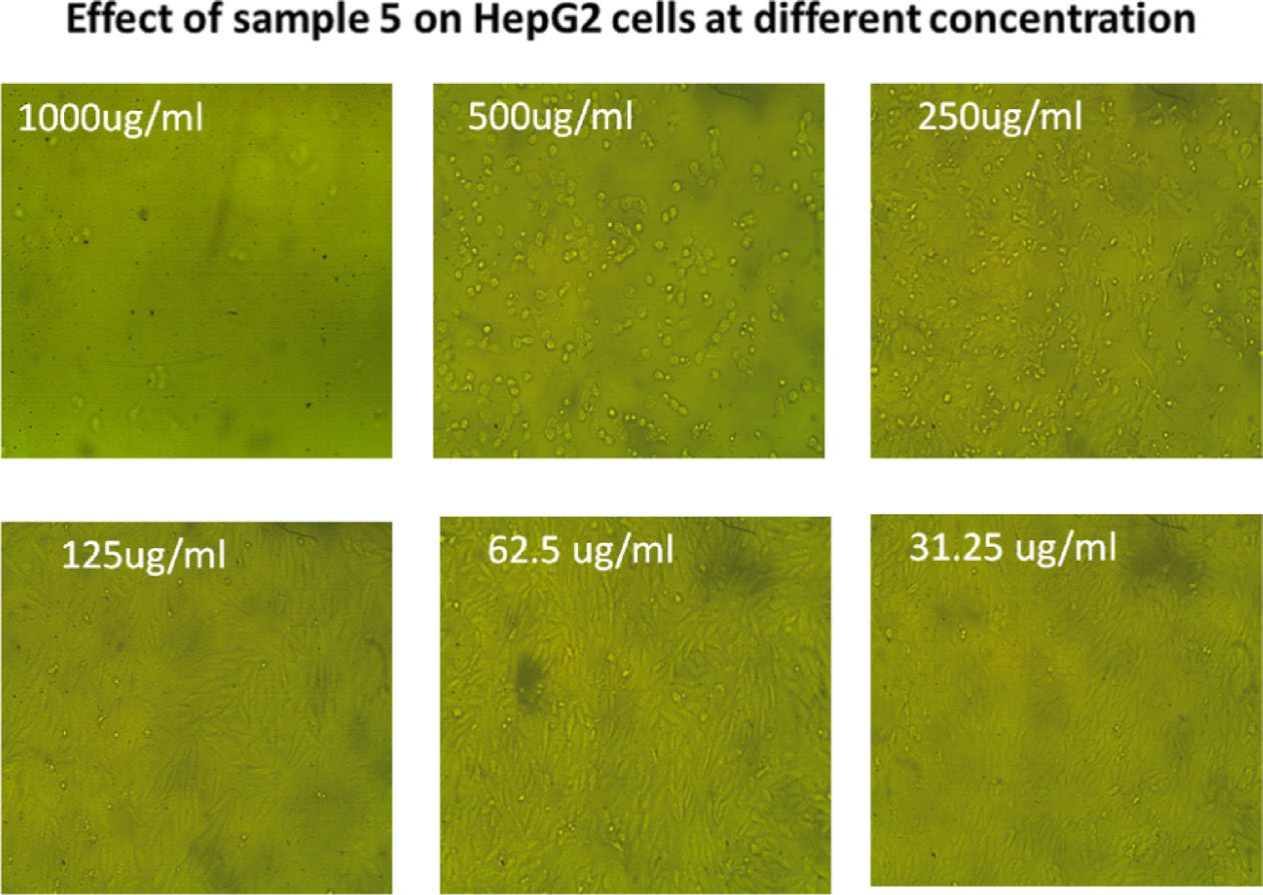
The effect of grapefruit peel extract on liver cell lines at different concentrations.

The anticancer activity of the ethanolic extract of grapefruit peels was evaluated against the HepG2 cell line using the MTT cytotoxicity assay. The results presented in Table 4; Figs. 1 and 2 demonstrate that the grapefruit peel extract (70% ethanol) exhibited a pronounced cytotoxic effect on HepG2 cells in a concentration-dependent manner.

At the highest tested concentration (1000 µg/mL), the extract reduced cell viability to 2.87%, corresponding to 97.13% cytotoxicity. A gradual decrease in cytotoxicity was observed with decreasing concentrations, confirming a dose–response relationship. The calculated IC₅₀ value of the extract was 230.63 ± 1.61 µg/mL, indicating moderate antiproliferative activity compared with the standard anticancer drug doxorubicin, which exhibited a significantly lower IC₅₀ value of 20.09 ± 2.54 µg/mL.

Doxorubicin showed consistently higher cytotoxicity across all tested concentrations, reflecting its greater potency. Statistical analysis (as indicated by different superscript letters) confirmed significant differences (*p* < 0.05) among the tested concentrations for both treatments.

**Table 4:** Anticancer activity (as % and IC_50_) of ethanolic extract of grapefruit peels against HepG2 cell line.

Concentration (µg/mL)	Plant ethanolic extract			Doxorubicin as an anticancer drug		
	Viability %	Toxicity%	IC_50_ (µg/mL) ± SD	Viability %	Toxicity %	IC_50_ (µg/mL) ± SD
1000	2.87	97.13^a^	230.63 ± 1.61	2.39	97.61^a^	20.09 ± 2.54
500	11.14	88.85^b^		2.63	97.37^a^	
250	42.92	57.0^c^		9.37	90.63^b^	
125	88.10	11.89^d^		16.69	83.31^c^	
62.5	99.82	0.1768^e^		30.99	69.01^d^	
31.25	99.82	0.1768^e^		49.55	50.45^e^	

The cytotoxic activity of grapefruit peel extract against HepG2 cells may be attributed to its high content of bioactive phytochemicals, particularly flavonoids, phenolic compounds, and limonoids (Table 5). These constituents are known to induce apoptosis through activation of mitochondrial pathways, including the upregulation of pro-apoptotic proteins and downregulation of anti-apoptotic proteins, leading to caspase activation. In addition, the extract may enhance the generation of reactive oxygen species (ROS), resulting in oxidative stress, mitochondrial dysfunction, and subsequent cancer cell death^40^.

Furthermore, the extract may inhibit cancer cell proliferation by inducing cell cycle arrest at critical phases such as G0/G1 or G2/M through modulation of cell cycle regulatory proteins. It may also interfere with key signaling pathways involved in cell survival and proliferation, including PI3K/Akt and MAPK pathways. These combined effects contribute to the suppression of tumor cell growth and may also reduce angiogenesis and metastatic potential, thereby enhancing the overall anticancer efficacy of the extract^41^.

### Chemical identification of active ingredients in grapefruit peel extract

#### Thin-layer chromatography, TLC

**Fig. 3:**
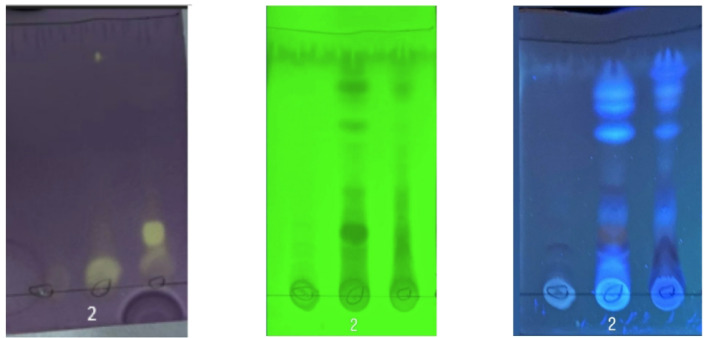
TLC chromatogram of grapefruit peel extracts, Spot number 2 and fraction (under short UV lamp at 254 nm) and TLC-bioautography of various extracts (Sprayed with DPPH reagent).

In many investigations, the ability to separate a mixture into its constituent chemical components is critical, allowing for the isolation of a specific product or the analysis of the mixture’s purity. Because of its low cost, simplicity, rapid development time, great sensitivity, and remarkable repeatability, thin-layer chromatography (TLC) is one of the simplest and most adaptable techniques for achieving this^42^. TLC is a flexible separation technique that is commonly used for qualitative as well as quantitative sample analysis. Data in Fig. 3 shows the TLC chromatogram of Grapefruit peel extracts. Spot number 2 and other fractions appeared using a short UV lamp at 254 nm and TLC-bioautography of various extracts after spraying with DPPH reagent. The results also showed that there was a strong separation of the compounds in the extract, which indicates high scavenging and antioxidant activity of grapefruit peels.

### HPlc analysis

**Fig. 4:**
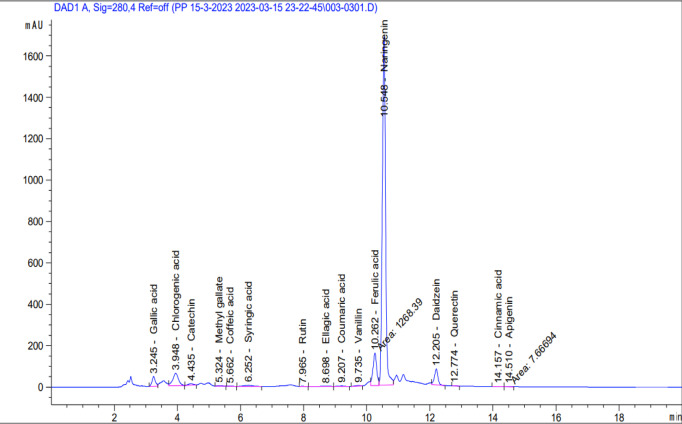
HPLC chromatogram of Ethanol extract from Grapefruit peels.

The high-pressure pumping of a sample (analyte) dissolved in a solvent (mobile phase) through a column containing an immobilized chromatographic packing material (stationary phase) is a type of column chromatography known as HPLC. The analytes’ retention time, or the rate at which they move through the column, is determined by the characteristics of the stationary phase, sample, and solvent^43^. In Table 5, the HPLC results in Fig. 4 demonstrated the presence of a total of 16 compounds. The highest concentration of the compounds was naringenin at 1444.62 µg/mL, followed by Chlorogenic acid with a concentration equal to 101.35 µg/mL and Ferulic acid came after them at 86.66 µg/mL, while the lowest concentration was shown at Cinnamic acid at 0.10 µg/mL, Caffeic acid at 0.28 µg/mL, Apigenin by 0.58 µg/mL, Quercetin by 0.88 µg/mL and Vanillin by 0.95 µg/mL.

These results agreed with those obtained by Choi et al.^10^, who showed that grapefruits are known to be rich in naringenin. Their study showed that naringenin has anticancer effects by inducing tumor cell death and inhibiting angiogenesis in malignant melanoma, indicating that naringenin has the potential to be a safe and effective treatment drug for melanoma. Also, the study of Banjerdpongchai et al.^44^ proved that naringenin, which is found in *Citrus* plants and seeds, causes apoptosis in human hepatocellular carcinoma HepG2 cells *via* mitochondria-mediated activation of caspase-9 and caspase-8-mediated proteolysis of Bid. The anticancer effects of the fruit peel extracts may be due to their high contents of phenolic and flavonoid compounds as well as plant pigments. In this regard, Xi et al.^45^ showed that gallic acid was the most abundant phenolic acid in all grapefruit examined, and Jiwei juice vesicles had the greatest gallic acid level (343.7 g/g DW). In the present study, the HPLC analysis of grapefruit peel extracts showed that gallic acid has a promising content (29.79 µg/mL), which supports the biological effect of the extract. (Table 5).

**Table 5:** List of phytochemical constituents (as µg/g) in ethanol extract of Grapefruit peels analyzed by HPLC.

Peak No.	Retention time (min)	Chemical name	Chemical structure	Conc. (µg/mL)	Biological activities	References
1	3.245	Gallic Acid		29.79	Antioxidant Anticancer Anti-inflammation	^46^
2	3.948	Chlorogenic acid		101.35	Anticancer Antioxidant	^46^
**3**	**4.435**	**Catechin**		**17.65**	**Anticancer** **Antioxidant**	^46^
**4**	**5.324**	**Methyl gallate**		**1.24**	**Anticancer** **Antioxidant**	^47^
**5**	**5.662**	**Caffeic acid**		**0.28**	**Anticancer** **Antioxidant**	^48^
**6**	**6.252**	**Syringic acid**		**6.70**	**Anticancer** **Antioxidant**	^49^
**7**	**7.965**	**Rutin**		**1.22**	**Anticancer** **Antioxidant**	^50,51^
**8**	**8.698**	**Ellagic acid**		**8.74**	**Anticancer** **Antioxidant**	^52,53^
**9**	**9.207**	**Coumaric acid**		**1.34**	**Anticancer** **Antioxidant**	^54^
**10**	**9.735**	**Vanillin**		**0.95**	**Anticancer** **Antioxidant**	^55^
**11**	**10.262**	**Ferulic acid**		**86.66**	**Anticancer** **Antioxidant**	^56,57^
**12**	**10.548**	**Naringenin**		**1444.62**	**Anticancer** **Antioxidant**	^10,58^
**13**	**12.205**	**Daidzein**		**36.65**	**Anticancer** **Antioxidant**	^59^
**14**	**12.774**	**Quercetin**		**0.88**	**Anticancer** **Antioxidant**	^60^
**15**	**14.157**	**Cinnamic acid**		**0.10**	**Anticancer** **Antioxidant**	^61^
**16**	**14.510**	**Apigenin**		**0.58**	**Anticancer** **Antioxidant**	^62^

### Molecular docking

#### Active site characterization

PrankWeb analysis of the AFP (7YIM) protein structure identified a well-defined binding cavity located within the hydrophobic interior of the protein, surrounded by a combination of aromatic, polar, and charged residues. The predicted active site encompassed key residues including LYS A:228, PHE A:172, CYS A:224, ALA A:225, GLU A:489, and MET A:490, among others. The diversity of chemical character among these residues — including the positively charged lysine, the aromatic phenylalanine, the thiol-bearing cysteine, and the acidic glutamate — suggests that the binding pocket is capable of accommodating a wide range of chemical interactions, including hydrogen bonding, electrostatic, hydrophobic, and pi-stacking contacts. This structural heterogeneity within the binding site provides a rational basis for the differential binding behavior observed among the tested phytochemicals.

#### Molecular docking result

The molecular docking results for all sixteen phytochemicals docked against AFP (7YIM) are summarized in Table 6. Binding affinities ranged from − 5.5 kcal/mol for vanillin to −8.9 kcal/mol for ellagic acid, reflecting substantial variability in the capacity of these structurally diverse molecules to complement the AFP active site.

**Table 6:** Binding affinities of sixteen phytochemicals docked against Alpha-fetoprotein (PDB ID: 7YIM).

Chemical Name (from HPLC table)	Binding Affinity (kcal/mol)
Gallic Acid	−6.7
Chlorogenic acid	−8.1
Catechin	−8.1
Methyl gallate	−6.0
Caffeic acid	−6.2
Syringic acid	−5.6
Rutin	−8.8
Ellagic acid	−8.9
Coumaric acid	−6.0
Vanillin	−5.5
Ferulic acid	−6.4
Naringenin	−8.4
Daidzein	−7.3
Quercetin	−8.5
Cinnamic acid	−6.2
Apigenin	−8.3

Ellagic acid emerged as the most potent binder with a binding affinity of −8.9 kcal/mol, a result consistent with its well-documented ability to form multiple simultaneous hydrogen bonds and pi-stacking interactions owing to its planar, polycyclic aromatic architecture and the presence of four hydroxyl groups. Rutin followed closely with an affinity of −8.8 kcal/mol; despite its larger molecular size, the sugar moiety attached to the flavonoid core appears to facilitate additional interactions with polar residues at the periphery of the binding site, compensating for potential entropic penalties associated with binding of a flexible molecule. Quercetin and naringenin, both flavonoids, achieved affinities of −8.5 and − 8.4 kcal/mol, respectively, underscoring the general suitability of the flavonoid scaffold for AFP binding. Apigenin and chlorogenic acid both achieved − 8.3 and − 8.1 kcal/mol, respectively, further supporting the notion that polyphenolic compounds with multiple hydroxyl groups and aromatic ring systems are structurally predisposed to engage productively with the AFP active site.

At the lower end of the binding affinity spectrum, simpler phenolic acids such as vanillin (−5.5 kcal/mol), syringic acid (−5.6 kcal/mol), methyl gallate (−6.0 kcal/mol), and coumaric acid (−6.0 kcal/mol) demonstrated comparatively weaker binding. This trend can be attributed to their smaller molecular volumes, fewer hydrogen bond donor and acceptor groups, and reduced capacity for pi-stacking, collectively resulting in less comprehensive occupation of the available binding cavity and fewer stabilizing contacts with active site residues.

### Protein-ligand interaction analysis

**Fig. 5:**
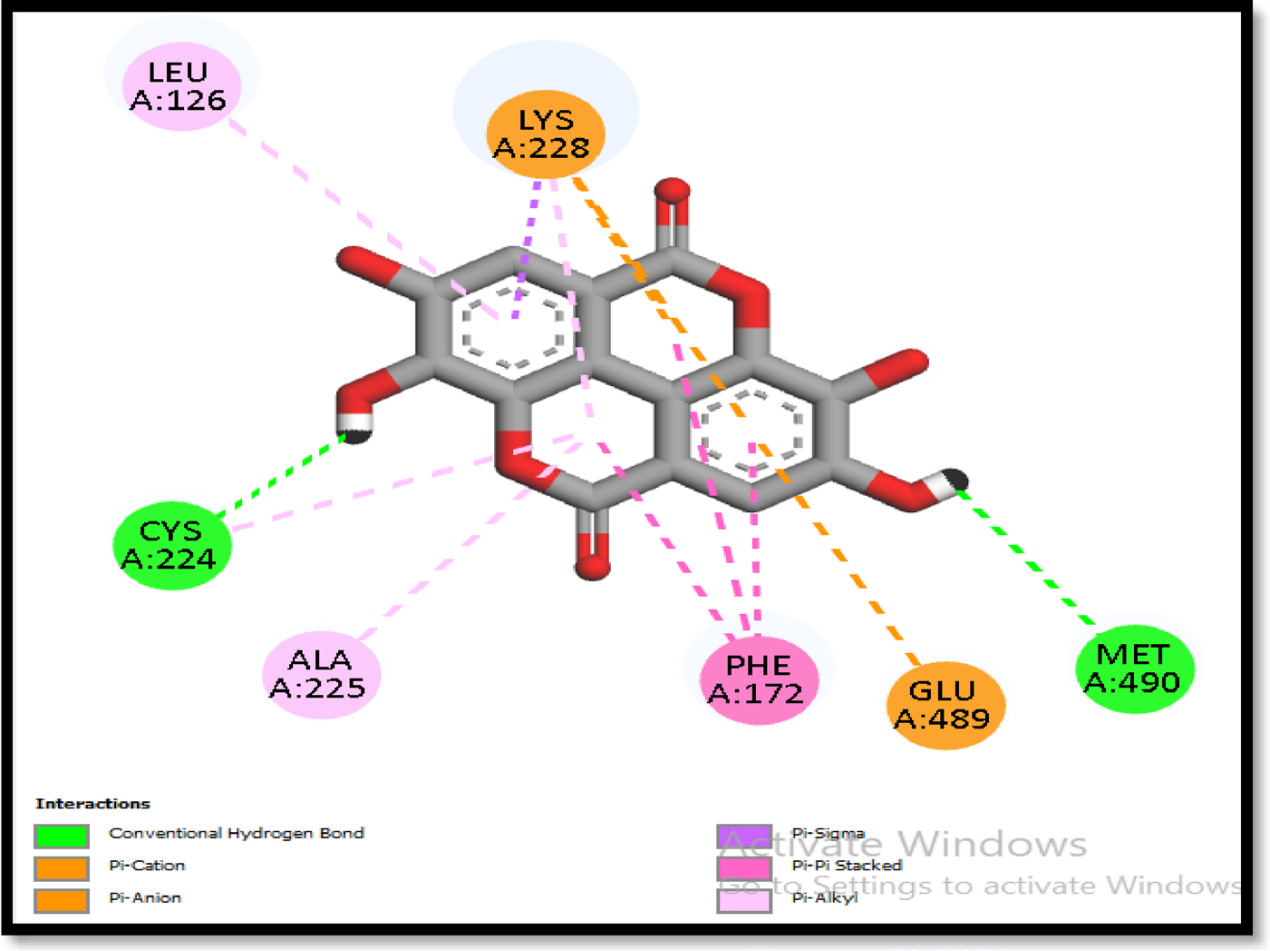
The 2D interaction map generated from the docking visualization for the ellagic acid as most promising compound with AFP binding pocket.

Detailed analysis of the protein-ligand interaction profiles for the highest-affinity compounds, conducted using Discovery Studio Visualizer, revealed that the top-ranked phytochemicals exploit a characteristic set of interactions within the AFP binding pocket. As illustrated in the interaction diagram for ellagic acid, the compound engages in a network of conventional hydrogen bonds mediated primarily through its hydroxyl substituents, which form contacts with the backbone and side chain atoms of key residues. Pi-pi stacking interactions were observed between the aromatic ring system of the ligand and the aromatic side chain of PHE A:172, providing substantial enthalpic stabilization to the complex. Pi-cation interactions with LYS A:228, as well as hydrophobic contacts involving MET A:490 and ALA A:225, further contributed to the overall binding stability (Fig. 5).

The interaction pattern observed for ellagic acid — involving LYS A:228, CYS A:224, PHE A:172, ALA A:225, GLU A:489, and MET A:490 — is consistent with the 2D interaction map generated from the docking visualization shown in Fig. 1. The pi-cation interaction with LYS A:228 (orange dashed lines) reflects the electrostatic complementarity between the electron-rich aromatic system of the ligand and the positively charged ammonium group of lysine. The pi-pi stacking with PHE A:172 (pink dashed lines) is characteristic of polycyclic aromatic compounds and contributes significantly to binding specificity. Hydrogen bond interactions (green dashed lines) with CYS A:224 and GLU A:489 provide directional stabilization, while van der Waals contacts with MET A:490 contribute to the hydrophobic component of binding. The pi-sigma and pi-alkyl interactions (purple and light pink dashed lines) involving the aliphatic portions of surrounding residues further reinforce the ligand within the cavity.

Quercetin and apigenin, both characterized by the 2-phenylchromen-4-one flavonoid core, displayed interaction profiles broadly similar to ellagic acid, with conserved pi-stacking engagement of PHE A:172 and hydrogen bonding with polar residues. The hydroxyl substitution pattern on the B-ring of flavonoids appears to be a critical determinant of both binding affinity and interaction profile specificity. Rutin, the glycosylated derivative of quercetin, exhibited an extended interaction network in which the rutinose sugar moiety projected toward the solvent-exposed edge of the binding site and established additional polar contacts, explaining its high binding affinity despite the entropic cost of immobilizing its flexible glycosidic linkages.

#### Comparison with simple phenolic compounds

The markedly lower binding affinities recorded for simple phenolic acids — including gallic acid (−6.7 kcal/mol), caffeic acid (−6.2 kcal/mol), cinnamic acid (−6.2 kcal/mol), and ferulic acid (−6.4 kcal/mol) — relative to the polycyclic flavonoids and tannin derivatives highlight the importance of molecular architecture in determining binding competence. While these monocyclic phenolic acids can participate in hydrogen bonding through their carboxylate and hydroxyl groups, they lack the extended aromatic surface area required for pi-stacking interactions with PHE A:172, and their smaller volumes result in incomplete occupation of the binding pocket, leaving hydrophobic sub-pockets unsatisfied. These structural limitations are reflected directly in their reduced binding affinity scores.

Nevertheless, it is worth noting that lower binding affinity does not preclude biological relevance; simpler compounds may offer advantages in terms of bioavailability, metabolic stability, and pharmacokinetic properties that partially compensate for their reduced binding potency. Future multi-parameter optimization studies should consider not only docking scores but also predicted ADMET (absorption, distribution, metabolism, excretion, and toxicity) profiles to identify the most drug-like candidates.

## Conclusion

In this study, the anticancer activity of ethanolic extract from grapefruit peels against the HepG2 cell line and the identification of the active ingredients in grapefruit peel extract were evaluated. The results showed that the extract had a variety of bioactive compounds that can work as antioxidants with high free radical scavenging activity and anticancer activities. In the molecular docking then naringenin is shown to inhibit tyrosine kinase, which may inhibit cancer cell proliferation and tumor progression. HPLC results show high concentrations of naringenin in grapefruit peels, and many studies have shown that it has anticancer properties and could induce tumor apoptosis in the HepG2 cell line. The molecular docking study identified ellagic acid, rutin, quercetin, and naringenin as the most promising AFP inhibitors, exhibiting strong binding affinities and stable interactions with key active site residues of the AFP protein. Among them, ellagic acid and rutin emerged as potential lead compounds for AFP-targeted therapy against hepatocellular carcinoma. However, further experimental validation, including in vitro, in vivo, and molecular dynamics studies, is necessary to confirm their therapeutic efficacy and binding stability.

Therefore, from the results, it could be concluded that grapefruit peels have therapeutic values and can be a promising natural drug against hepatocellular carcinoma. However, it is recommended in this study that working in vivo (using experimental animals) can more accurately assess the effectiveness, safety, and toxicity of the plants.

### Study limitations and future perspectives

The present study is limited by its in vitro experimental design using the HepG2 hepatocellular carcinoma cell line, which does not fully reflect the complexity of in vivo biological systems. Therefore, the observed anticancer effects of grapefruit peel extract may not directly translate to physiological conditions in living organisms. In addition, the study lacks in vivo animal model validation and clinical evidence to confirm its safety, bioavailability, and therapeutic efficacy. Future investigations are recommended to include in vivo studies and mechanistic evaluations to further elucidate the pharmacokinetics, toxicity profile, and molecular targets of grapefruit peel extract, thereby supporting its potential development as a natural anticancer agent.

## Data Availability

The authors declare that the data supporting the findings of this study are available within the paper. Should any raw data files be required in an alternative format, they are available from the corresponding author upon reasonable request.
